# Posttranslational regulation of the bile salt export pump

**DOI:** 10.1186/2047-783X-19-S1-S20

**Published:** 2014-06-19

**Authors:** Susanne Przybylla, Lutz Schmitt

**Affiliations:** 1 Institute of Biochemistry, Heinrich Heine University, 40225 Düsseldorf, Germany

## 

Bile plays an essential role in the nutrient uptake of the human body. It consists mostly of amphipathic bile salts, phospholipids and cholesterol in mixed micelles. The bile solubilizes lipophilic nutrients, such as lipids and fat-soluble vitamins, and facilitates their uptake. The formation of bile depends on the activity of various ATP-binding cassette (ABC) transporters localized in the apical membrane of hepatocytes. The main components of bile, phosphatidylcholine, cholesterol and bile salts, are transported by multidrug resistance protein 3 (MDR3, ABCB4), ABCG5/G8 and the bile salt export pump (BSEP, ABCB11), respectively. BSEP, as the transporter of the main solute in bile, is considered to be the primary driving force of bile formation.

BSEP belongs to the family of ABC transporters, which constitute one of the largest protein families. They are primary active transporters with a common functional unit: two nucleotide–binding domains and two transmembrane domains. The nucleotide–binding domains fuel the transport by hydrolyzing ATP, while the transmembrane domains provide the translocation pathway over the membrane and are thought to be responsible for substrate recognition. Eukaryotic ABC transporters are usually encoded by one gene as in the case of BSEP (full-size transporters) or by two genes coding for one nucleotide-binding domain and one transmembrane domain each. In the latter case the transporter is called a half-size transporter and the two polypeptides homo- or heterodimerize to form a functional unit. An example for such a half-size transporter is ABCG5/G8. Mutations in ABC transporters can lead to severe diseases. For example an impairment of targeting or activity in BSEP leads to the accumulation of bile salts in the hepatocytes. Bile salts, in addition to their detergent function, can furthermore act as signaling molecules and thereby cause different forms of cholestasis, such as progressive familial intrahepatic cholestasis type 2 (PFIC2) or benign recurrent intrahepatic cholestasis type 2 (BRIC2).

An additional influence that can affect the development of diseases is the post-translational regulation of ABC transporter functionality. The regulation can occur directly in the form of chemical modifications or can be mediated for example by protein-protein interactions. For several ABC transporters of the apical hepatocyte membrane an influence of protein-protein interactions on targeting, turnover or activity could be shown.

MRP2 for example interacts with NHERF-1, a scaffold protein that is responsible for linking its interacting partners to the cytoskeleton. It has furthermore been reported that the interaction of MRP2 with NHERF-1 influences the apical membrane expression of MRP2. For example, NHERF-1 knock–out mice show reduced abundance of MRP2 in the membrane [1].

For BSEP two interaction partners are known to date. One of them is the AP2 adaptor related protein complex, which is involved in internalization of BSEP [2].

To study ABC transporters in their isolated form a heterologous overexpression system is required to obtain sufficient amounts of protein. Initially, toxicity of the BSEP cDNA for *Escherichia coli* was an obstacle. Therefore a *Saccharomyces cerevisiae* based cloning technique has been developed, which is independent of *E. coli*[3]. Subsequently, with the optimized cloning procedure an overexpression of BSEP could be established in the methylotrophic yeast *Pichia pastoris*. With the help of a GFP-fusion protein BSEP could be traced to the *P. pastoris* plasma membrane. Additionally, sucrose density centrifugation experiments confirmed the correct localization of BSEP. A subsequent detergent screen led to the identification of detergents suitable for BSEP solubilization. The transporter appears to be in a detergent-resistant environment as only the lipid–like, zwitter–ionic detergents of the Fos–choline and Cyclofos series, comparatively “harsh” detergents, could efficiently extract BSEP. Finally, a purification protocol was established to obtain BSEP in a detergent-solubilized form. With a dual–affinity tag tandem affinity purification could be employed to obtain the ABC transporter with approximately 75% purity [4].

In summary, the optimized cloning strategy for BSEP cDNA in *S. cerevisiae* as well as the efficient expression and purification protocols enable us to investigate the interaction of BSEP with other proteins *in vitro*.
